# Lessons to Be Learned From the COVID-19 Pandemic: Some Further Ideas

**DOI:** 10.3389/ijph.2025.1609023

**Published:** 2026-02-02

**Authors:** Alberto Donzelli

**Affiliations:** Allineare Sanità e Salute Foundation, Milan, Italy

**Keywords:** COVID-19 lockdown, COVID-19 vaccine myo-pericarditis, vaccinated vs. unvaccinated mortality, infection and transmission, boosters and SARS-CoV-2 infections

Quinn et al. [1] have done an exceptional work. I offer some further suggestion.

The Authors state:

(*page 8*) “the progression of the pandemic was largely independent of government measures”. Indeed, some government measures were even counterproductive for hard outcomes. For example, all-cause mortality excess in 2020 (pre-vaccination era) versus mean mortality 2015–2019 (Our World in Data) was not favorably associated with the lockdown index (Oxford University Database). Rather, it was slightly unfavorably associated with mortality both globally, and in subanalysis by gross domestic product *per capita* ($ <21,000; 21,000 to <45,000; ≥45,000), population size (<17; 17 to 50, >50 M), population density (inhabitants per km^2^: <100, 100 to <1,000, ≥1,000), and proportion of older adults (75–84, or ≥85 years).

(*page 15*) “the incidence of… transient myocarditis and/or pericarditis is not ‘rare’, but rather ‘uncommon’”. The correct term is ‘common’ (i.e. 2.33%–2.8% in the two cited [1] studies, as per international rules).

(*page 16*) “some studies comparing all-cause mortalities between vaccinated and unvaccinated groups found slightly increased risks among vaccinated groups”. Indeed, in the cited study, in the 2 years 2021 and 2022, the mortality risk in vaccinated people with one or two doses was statistically significant, and twice or more than the risk of unvaccinated. Moreover, a study based on the UK Office for National Statistics (ONS) data [2] shows that the regression lines, both for all-cause deaths and for non-COVID-19 deaths, start from very low values for all the age groups, with almost linear progressive increases as the months progress. For the age groups 18–39, 80–89 and 90+ years, both regression lines intersect and overcome the reference line of unvaccinated during the study period. The same occurs for the age 50–59, limited to non-COVID-19 deaths. For the other age groups, the month of the intersection was predicted calculating the angular coefficients. The last intersections would have occurred in 2024, but the ONS stopped publishing mortality data by vaccination status after May 2023.

(*page 17*) “by late 2021, it was abundantly clear that the vaccines did not prevent COVID-19 infection”. Indeed, by March 2022 it was clear that, in the Delta variant era, they did not prevent even the transmission; instead, they significantly increase the transmission from infected people, ≥90 days after dose 2 [3].

Moreover, many recent studies (e.g., [4, 5]) showed that multiple inoculations are progressively and proportionally associated with SARS-CoV-2 infections.
